# Investigation of Self-Healing Mortars with and without Bagasse Ash at Pre- and Post-Crack Times

**DOI:** 10.3390/ma15051650

**Published:** 2022-02-23

**Authors:** Belay Brehane Tesfamariam, Redeat Seyoum, Dinsefa Mensur Andoshe, Tatek Temesgen Terfasa, Gulam Mohammed Sayeed Ahmed, Irfan Anjum Badruddin, H.M.T. Khaleed

**Affiliations:** 1 Department of Materials Science and Engineering, Adama Science and Technology University, Adama 1888, Ethiopia; redeatseyoumseyoum@gmail.com (R.S.); dinsefadear@gmail.com (D.M.A.); 2 Department of Chemical Engineering, Adama Science and Technology University, Adama 1888, Ethiopia; tate95et@gmail.com; 3 Program of Mechanical Design and Manufacturing Engineering, School of Mechanical, Chemical and Materials Engineering, (So-M-C-M-E), Adama Science and Technology University, Adama 1888, Ethiopia; gmsayeed.ahmed@astu.edu.et; 4 Center of Excellence (COE) for Advanced Manufacturing Engineering, Program of Mechanical Design and Manufacturing Engineering, School of Mechanical, Chemical and Materials Engineering, (So-M-C-M-E), Adama Science and Technology University, Adama 1888, Ethiopia; 5 Research Center for Advanced Materials Science (RCAMS), King Khalid University, Asir 61413, Saudi Arabia; irfan@kku.edu.sa; 6 Mechanical Engineering Department, College of Engineering, King Khalid University, Abha 61421, Saudi Arabia; 7 Department of Mechanical Engineering, Faculty of Engineering, Islamic University of Madinah, Madinah Munawara 42351, Saudi Arabia; khalid_tan@yahoo.com

**Keywords:** mortars, self-healing, *Bacillus subtilus*, calcium lactate, bagasse ash

## Abstract

Cracks in typical mortar constructions enhance water permeability and degrade ions into the structure, resulting in decreased mortar durability and strength. In this study, mortar samples are created that self-healed their cracks by precipitating calcium carbonate into them. *Bacillus subtilus* bacterium (10^−7^, 10^−9^ cells/mL), calcium lactate, fine aggregate, OPC-cement, water, and bagasse ash were used to make self-healing mortar samples. Calcium lactates were prepared from discarded eggshells and lactic acid to reduce the cost of self-healing mortars, and 5% control burnt bagasse ash was also employed as an OPC-cement alternative. In the presence of moisture, the bacterial spores in mortars become active and begin to feed the nutrient (calcium lactate). The calcium carbonate precipitates and plugs the fracture. Our experimental results demonstrated that cracks in self-healing mortars containing bagasse ash were largely healed after 3 days of curing, but this did not occur in conventional mortar samples. Cracks up to 0.6 mm in self-healing mortars were filled with calcite using 10^−7^ and 10^−9^ cell/mL bacteria concentrations. Images from an optical microscope, X-ray Diffraction (XRD), and a scanning electron microscope (SEM) were used to confirm the production of calcite in fractures. Furthermore, throughout the pre- and post-crack-development stages, self-healing mortars have higher compressive strength than conventional mortars. The precipitated calcium carbonates were primed to compact the samples by filling the void spaces in hardened mortar samples. When fissures developed in hardened mortars, bacteria became active in the presence of moisture, causing calcite to precipitate and fill the cracks. The compressive strength and flexural strength of self-healing mortar samples are higher than conventional mortars before cracks develop in the samples. After the healing process of the broken mortar parts (due to cracking), self-healing mortars containing 5% bagasse ash withstand a certain load and have greater flexural strength (100 kPa) than conventional mortars (zero kPa) at 28 days of cure. Self-healing mortars absorb less water than typical mortar samples. Mortar samples containing 10^−7^ bacteria cells/mL exhibit greater compressive strength, flexural strength, and self-healing ability. XRD and SEM were used to analyze mortar samples with healed fractures. XRD, FTIR, and SEM images were also used to validate the produced calcium lactate. Furthermore, the durability of mortars was evaluated using DTA-TGA analysis and water absorption tests.

## 1. Introduction

Self-healing concretes and mortars are currently receiving a lot of attention, since they provide a longer service life to building projects by fixing fractures themselves. Mortars and concrete are often utilized in man-made building materials that are robust in compression but rather weak in tension. Concrete structures commonly develop cracks during the pre-hardening and post-hardening stages of the cement paste owing to plastic and dry shrinkage [1,2], thermal stresses [2,3], the settling process [4,5], freeze–thaw [6,7], and applied load [5]. The existence of fractures on concrete means water and air can easily enter into a concrete building. This causes the rebar to corrode, affecting the longevity and integrity of the entire concrete construction. Cracks also allowed hazardous ions such as chloride ions, sulphate ions, and CO_2_ to enter concrete constructions, causing strength loss and eventual structural collapse [8]. As a result, it is critical to limit fracture growth and repair cracks as soon as feasible to increase the life of the constructed structure. Manual grouting [9], surface treatment [10], route sealing [11], and injecting chemical adhesives [12] into fractures are approaches for mending concrete cracks. However, these methods require continuing maintenance and are costly; also, some of the chemical adhesives are harmful to the environment. In previous work done [13], it was also described autonomous approaches for concrete self-healing. Self-healing concrete may cure cracks on its own, preserving the internal matrix as well as the reinforcement steel and resulting in a longer service life [14]. Self-healing materials are artificial or synthetically generated substances that have the built-in ability to autonomously mend defects (cracks) without the need for external aid or human involvement. In the chemical self-healing approach, chemical liquid reagents or glues are injected into fractures, or these chemical reagents/glues are placed in a capsule and then placed in concrete before it hardens [15,16,17]. However, the chemical self-healing process is costly, sophisticated, and makes the process of casting concrete is more difficult. As a result, it is important to choose a strategy for fixing concrete cracks that is cost-effective, has an easy casting process, is harmless to humans and the environment, and also effectively heals the cracks. The development of bio-concrete has recently received great attention due to its lower maintenance costs, efficacy in mending wide crack widths, the environmental friendliness of the healing agent, and also the definite bacteria that can survive in hard concrete settings. In bio-concrete, bacterial spores feed the nutrient (calcium lactate) in the presence of water and subsequently generate calcium carbonate to heal the fractures [18,19]. Furthermore, the bacteria consume oxygen present in concrete during their development, thus protecting the reinforcing steel bar from corrosion [16,18,19,20]. Thus, as mentioned in recent research [18], fracture repair utilizing innocuous bacteria is ecologically acceptable and has great compatibility with concrete materials. However, only a few bacteria types can withstand extreme concrete settings since it shortens their life span. Bacteria S. pasteurii or Bacillus pasteurii are often-utilized bacteria for self-healing concrete due to their endospores’ ability to withstand the harsh environment of concrete [21]. Furthermore, the inclusion of Bacillus pasteurii into concretes increased the compressive strength, durability, and resistance to freeze–thaw of samples due to calcite precipitation. Moreover, the water absorption and chloride permeability of bio-concrete reduced because of the precipitated calcite-filled cracks/voids [22,23]. Concrete compressive and flexural strengths were enhanced by 28% using Bacillus subtilis bacteria [21,23]. Similarly, the addition of Bacillus megaterium and Bacillus pasteurii (10^−5^ cells/mL) to concrete boosted its compressive and flexural strength up to 24% owing to the creation of calcite, which filled voids and prevented the entry of toxic chemicals into the structure [24,25,26]. A recent study has reported that the best self-healing concrete width utilizing Bacillus cohnii is 0.56 mm after 28 days of cure [27]. Self-healing concrete samples with the addition of Bacillus sphaericus at various concentrations were also evaluated, and their report revealed a concentration of bacteria 10^5^ cells/mL delivered superior compressive strengths to bio-concrete [28]. However, bio-concretes are more expensive than regular concrete due to the high price of calcium lactate (C_6_H_10_CaO_6_), and the technique of casting concrete. In the encapsulation method, bacteria and calcium lactate are encased in the capsule to protect the bacteria from a hostile environment. However, the casting of cement–concrete-containing capsules is laborious and expensive. In the direct approach process, a mix of bacteria solution and calcium lactate powder together with cement, aggregates, and water for the fabrication of concrete is simple and economical [29]. Thus, we employed the direct approach process for sample preparation and selected bacteria that were not harmful.

Therefore, our study focused on the investigation of self-healing mortars with and without the addition of bagasse ash at pre- and post-crack development times. We made Self-Healing Mortars (SHM) using harmless bacteria, Portland cement, and control-burnt bagasse ash, fine sand, calcium lactate powder, and water. Control-burnt bagasse ash was used as a partial replacement for Ordinary Portland Cement (OPC) since bagasse ash has increased pozzolanic reactivity, and also this replacement has economic benefits, as indicated in our earlier paper [30]. Calcium lactate powder was also prepared from discarded eggshells and lactic acid to reduce the material cost of SHM. Compressive strength, flexural strength, water absorption, thermal analysis, and microstructural analysis were all performed. Furthermore, the self-healing potential of mortars was tested using two different bacterial concentrations with and without the addition of bagasse ash.

## 2. Experimental Procedures

### 2.1. Preparation and Analysis of Calcium Lactate Powder and Bagasse Ash

Self-healing mortars (SHM) and concretes (SHC) are expensive, mainly due to the calcium lactate powder material and the production technique. We synthesized calcium lactate (C_6_H_10_CaO_6_) powder, and also prepared control-burnt bagasse ashes as OPC cement replacement to reduce the material cost of self-healing mortars. To make calcium lactate powder, leftover eggshells were cooked in distilled water for 2 h (to remove the white film within) and then dried for another 2 h. Following that, eggshells were pulverized and sieved to produce calcium carbonate powder. We combined 10 g CaCO_3_ powder with 20 cc lactic acid and stirred it for 2 h at 50 °C. The mixes were dried and pulverized after a 30 min filtering procedure to yield calcium lactate powder. Previous research [31,32] have reported that the addition of a high amount of calcium lactate in SHC reduces the compressive strength of concrete; thus, a tiny amount (1%) of calcium lactate was employed to make an SHM in our samples. We employed an X-ray Diffractometer (Shimadzu, XRD 7000, CuK = 1.5418), Scanning Electron Microscopy (SEM, Shimadzu, COXIEM-30), and Fourier Transform Infrared Spectroscopy (FTIR) to demonstrate the production of calcium lactate from discarded eggshell and lactic acids. We also prepared control-burnt bagasse ash (C-BA) by burning at 300 and 600 °C/2 h. Control-burnt bagasse ash was subsequently analyzed by Differential Thermal Analysis–Thermogravimetry Analysis (DTA-TGA) (Shimadzu, DTG-60H) and X-ray Diffractometer, as described in our earlier work. SEM images were also utilized to look at the microstructures of burnt bagasse ash at both a lower temperature of 300 °C/2 h and a higher temperature of 600 °C/2 h. In this study, we only used the control-burnt bagasse ash at 600 °C/2 h for sample preparation and we symbolized it as C-BA.

### 2.2. Preparation of Bacteria with Two Different Concentrations

The *Bacillus subtilus* bacterium was chosen for our investigation due to its high survival rate in an alkaline environment and ability to precipitate CaCO_3_. To make the bacterial culture medium, we dissolved 5.0 g of peptone and 3.0 g of beef extract in one liter of distilled water. After adjusting the pH of the culture medium to 7.0, it was sterilized at 121 °C for 20 min and chilled in the clean station. One milliliter (mL) of bacteria was collected from the broth culture and placed in 9 mL of distilled water test tubes to obtain 10^−1^ cells/mL, after which serial dilution was performed to obtain 10^−7^ and 10^−9^ cells/mL. For sample preparation, we employed bacteria at doses of 10^−7^ cells/mL (to have a higher amount of bacteria cells) and 10^−9^ cells/mL (for a relatively lower amount of bacteria).

### 2.3. Fabrication of Self-Healing Mortars with(out) Bagasse Ash for Analysis

Self-healing mortar (SHM) samples with composition (%) of Ordinary Portland Cement (100 − x)%, calcium lactate powder (x)%, *Bacillus subtilus* Bacteria (10^−7^ or 10^−9^ cells/mL), sand and water (W) were prepared by the conventional method at room temperature, with average humidity values ranging from 31–46%. The composition of calcium lactate powder (x%) was = 0 or 1; W/(Cement + x) ≈ 0.53 and cement to sand ratio was = 1:3. These prepared mortar bricks samples were cured for various curing ages at room temperature at 3 days, 7 days, 14 days, and 28 days, separately. In addition, we fabricated self-healing mortar samples containing 5% control burnt bagasse ashes (SHM + 5% C-BA) (Table 1). For the fabrication of SHM specimens, we employed two bacterial cell concentrations of 10^−7^ and 10^−9^ cells/mL. Specimen sizes of 70 mm × 70 mm × 70 mm were manufactured for compressive strength testing. Bacteria (10^−7^ or 10^−9^ cells/mL), 1% calcium lactate powder, 5% C-BA, 95% OPC-cement, sand, and water were used to fabricate mortar samples. We previously explored 5% C-BA-blended OPC-mortar samples that had a more advanced compressive strength than unblended OPC-mortar samples at early and late curing ages [30]. Thus, in this study also, we employed just 5% C-BA as an OPC-cement alternative to make SHM samples. After 3, 7, 14, and 28 days of curing the samples, compressive strength and water absorption tests were performed. XRD and SEM analyses were performed to check whether the precipitated powder inside the self-healing mortar fissure was calcite material or not. We also used DTA-TGA to assess the thermal stability of mortar samples.

**Figure 1:**
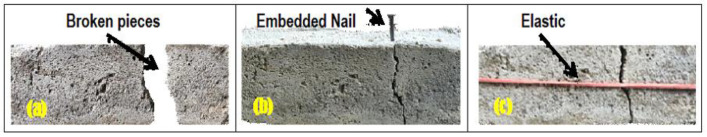
Artificial crack creation in mortar samples; (**a**) broken pieces; (**b**) Nail embedded in crack-creation setup; (**c**) post-crack before healing which was held using elastic.

To evaluate flexural strength as well as bacterial healing efficacy, mortar samples with dimensions of 160 mm × 50 mm × 50 mm were cast using bacteria (10^−7^ cells/mL), calcium lactate (1%), OPC-cement (95%), C-BA (5%), sand, and water. The crack-healed zone of the mortars was seen using optical microscopy (OP). After 3 days of curing the samples, artificial fractures of varying sizes (inserting different nails) were made in mortar samples, as shown in Figure 1a–c; and then the self-healing process was monitored after 7 and 14 days. The healed width of mortar samples was measured to determine the self-healing efficacy of mortar at the macro and micro levels. The three-point bending point technique was used to determine the flexural strengths of mortar samples. Table 2 displays the compositions of conventional mortar (CM), self-healing mortar (SHM), and self-healing mortar with bagasse ashes (SHM + C-BA) for the flexural strength test.

## 3. Results and Discussion

### 3.1. Characterization of Synthesized Calcium Lactate and Bagasse Ash

**Figure 2:**
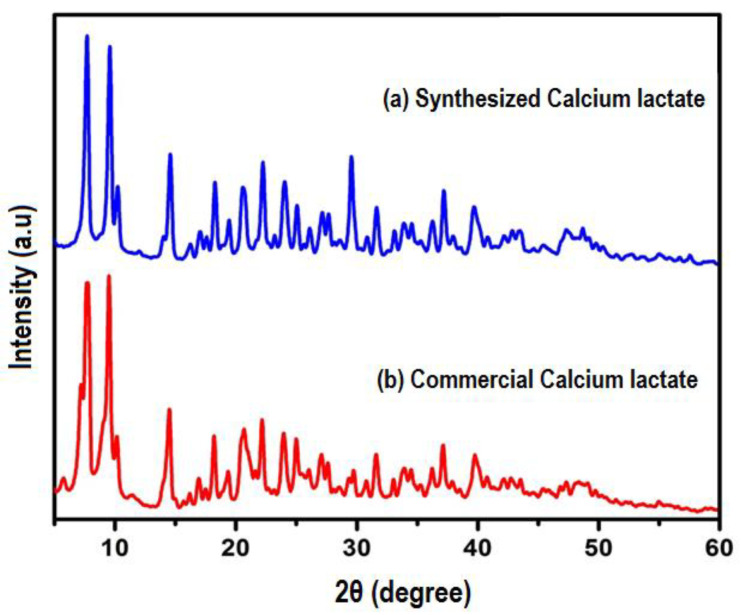
XRD pattern: (**a**) synthesized and (**b**) commercial (reference) calcium lactate powders.

An XRD analysis was used to validate the produced calcium lactate. Figure 2 indicates that the XRD peaks of synthetic calcium lactate and commercially manufactured calcium lactate are well-matched, with both powders exhibiting two main peaks at 7.8° and 9.6° on 2θ degree. A similar XRD finding was observed with calcium lactate, which is present in Cheddar cheese [33]. As a result, we were able to successfully synthesize calcium lactate from discarded eggshells and lactic acid.

**Figure 3:**
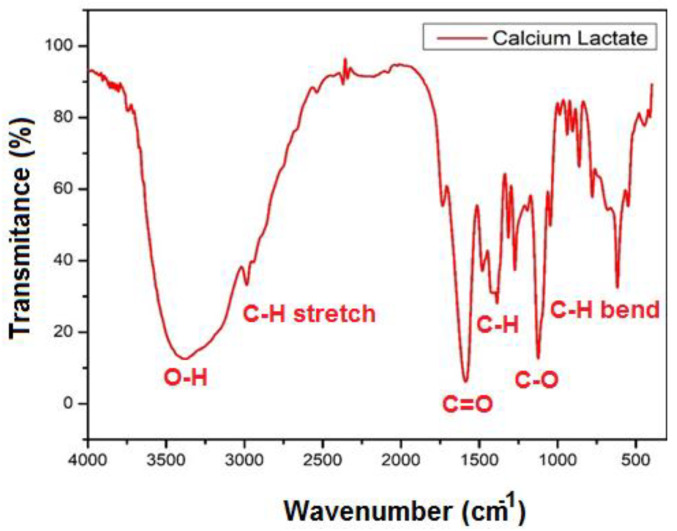
FTIR analysis of the synthesized calcium lactate powder.

The existence of functional groups (or chemical bonds) in the produced calcium lactate was also confirmed using Fourier-transform infrared spectroscopy (FTIR) (C_6_H_10_CaO_6_). The absorption band of calcium lactate emerged in the region of 2800 to 3500 cm^−1^ based on the FTIR results (CH stretch). At around 3000~3500 cm^−1^, a prominent OH valence band of calcium lactate emerged. The carbonyl band’s absorption peak is detected at 1500~1700 cm^−1^ (C=O stretching). Furthermore, we found absorption signals ranging from 1300 to 1400 cm^−1^ (CH-inflection point) (Figure 3). As a consequence, the FTIR data suggests that the interaction of CaCO_3_ from eggshells with lactic acid resulted in the formation of calcium lactate.

**Figure 4:**
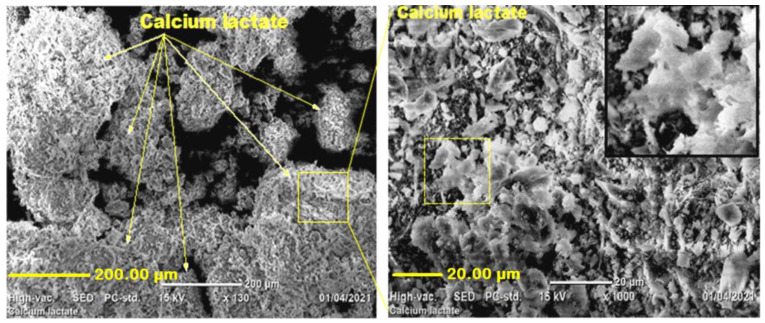
SEM images of the synthesized calcium lactate powder.

The microstructure of calcium lactate crystals, which was generated from 10 g of calcite and 20 mL of lactic acid at 50 °C, was also examined using SEM imaging. As seen in Figure 4, calcium lactate produces multilayer crystals with rough surfaces; plate-shaped calcium lactate particles adhere together and form massive aggregate particles (crystals). This crystal is an indication of the formation of calcium lactate. In prior work [29], calcium lactate, which was produced from commercially available aragonite, calcium carbonates, and lactic acid, has shown a similar microstructure crystal in SEM image.

**Figure 5:**
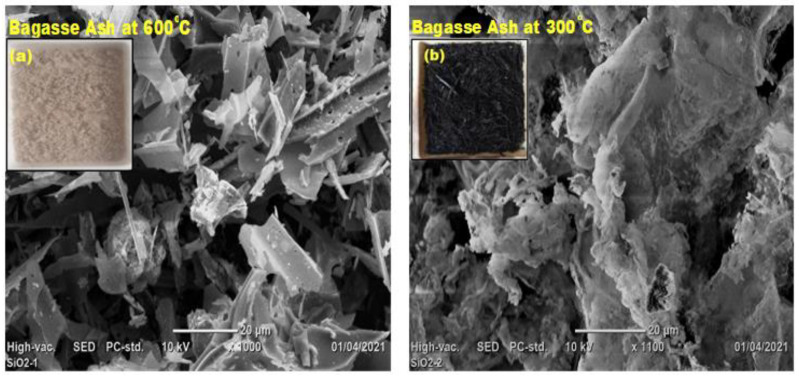
SEM images: (**a**) bagasse ash at 600 °C/2 h; (**b**) bagasse ash at 300 °C/2 h.

We burnt bagasse ash at 600 °C/2 h and used SEM images to examine its microstructure (Figure 5a). Control-burnt bagasse ash (C-BA) exhibits very porous microstructures and is white, as illustrated in the inset camera picture in Figure 5a. The white hue of C-BA indicates the carbon, and certain components/ions in BA most likely removed at a higher burning temperature of 600 °C. On the other hand, BA at 300 °C/2 h has less porous microstructures and is black, as illustrated in the inset camera image (Figure 5b). At a burning temperature of 300 °C/2 h, BA contains higher carbon and it reduces the strength of BA-blended mortars. Compressive strength BA-blended OPC-mortars increasing with a carbon concentration of the bagasse ash decreases, according to earlier research [30,34]. As a result, we created BA-blend self-healing mortar samples by burning BA at 600 °C/2 h.

### 3.2. Visual Inspection of Cracks

(i) Self-Healing Mortars at post-crack development

**Figure 6:**
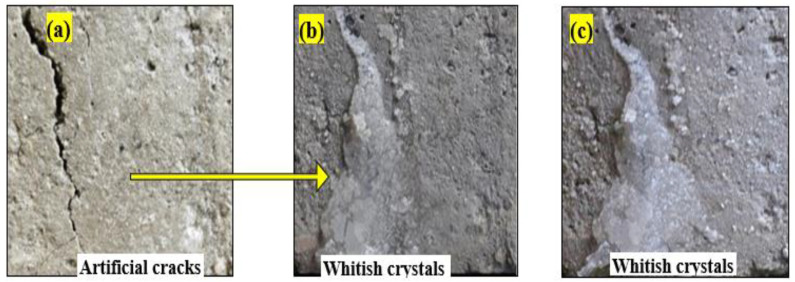
Self-Healing Mortars (SHM) containing bacteria concentration of 10^−7^ cell/mL: (**a**) before healing; (**b**) healing after 7 days’ curing; (**c**) healing after 14 days’ curing.

**Figure 7:**
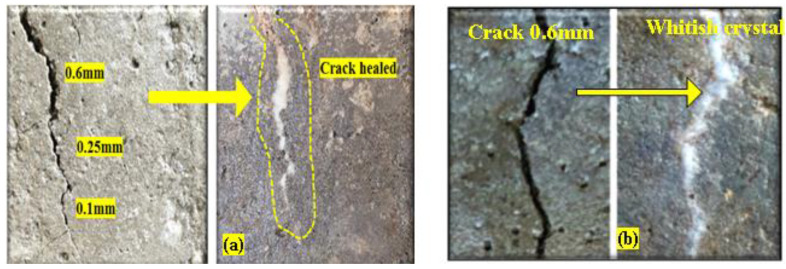
Healed cracks on Self-Healing Mortars with bacteria: (**a**) 10^−7^ cells/mL; (**b**) 10^−9^ cells/mL.

Figure 6 and Figure 7 show that fracture widths of up to 0.6 mm were entirely sealed for SHM samples. As previously mentioned [18], bacteria become dormant and enter the dormancy stage when specimens harden, and then activate in the presence of moisture or water and feed calcium lactate to generate calcium carbonate, which closes the fissures. Cracks in mortar samples having bacterium densities of 10^–7^ cells/mL were filled after 7 and 14 days of curing, as shown in Figure 6a–c. Excess white crystals were seen precipitating along the fissures.

Similarly, cracks on mortars containing bacteria 10^−9^ cells/mL were filled by calcite after 7 days of curing ages (Figure 7b). Moreover, we observed that self-healing efficiency also increased with increasing bacterial concentration and curing duration.

(ii) Self-Healing Mortars with Bagasse ashes at post-crack development

**Figure 8:**
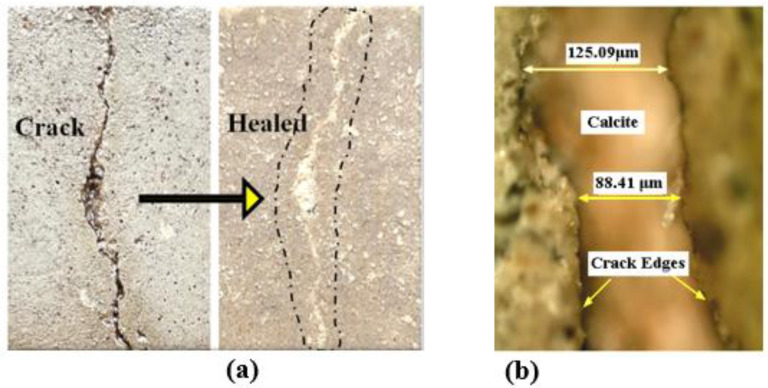
(**a**) Healed crack on Self-Healing Mortar contained 5% control burned BA and bacteria 10^−7^ cell/mL; (**b**) optical microscope image (×50 magnification) of SHM.

Figure 8a,b depicts the healing of cracks up to 0.2 mm in width of a Self-Healing Mortar sample containing 5% BA after 7 days of curing. Figure 8a shows excessive white crystals formed along the fracture area. However, we found that the fracture healing effectiveness of mortars reduced as BA replacement increased, as did the usage of BA burnt at 300 °C. Optical microscope pictures (×50 magnification) reveal that the precipitated calcite crystalline fills fracture locations on the mortar samples (Figure 8b).

### 3.3. X-ray Diffraction (XRD) Analysis of the Precipitated Whitish c=Crystal

**Figure 9:**
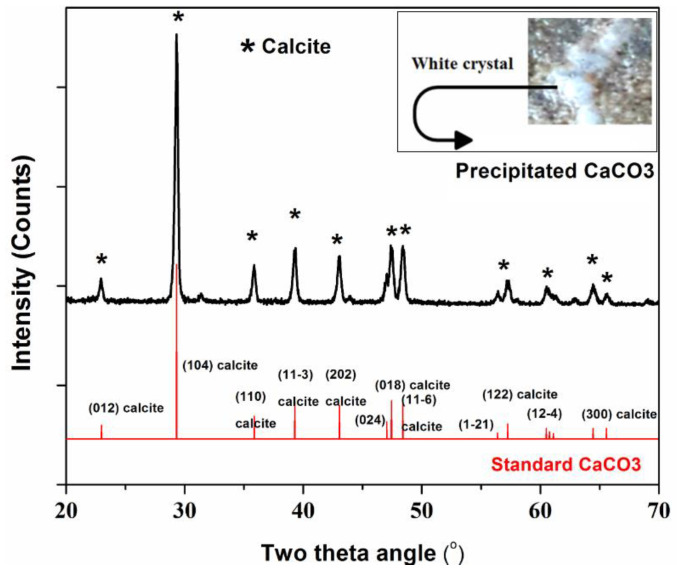
XRD image of the precipitated Calcite (CaCO_3_) by bacteria.

We measured the XRD of the precipitated white crystal powders from the fracture area. X-ray diffraction patterns (Figure 9) revealed several diffraction peaks at a 2θ angle for both reference calcite (CaCO_3_) and precipitated white powders. The precipitated white powders’ peaks are well-matched with reference calcite material, calcite crystal, (JCPDS NO. 00-047-1743). Thus, the precipitated powder along the fracture surface of mortar is predominantly phased for calcite, indicating that the bacillus bacteria successfully produced calcite to repair the mortar break. As seen in the inset picture in Figure 9, the XRD result confirmed that the precipitated white crystal along the fracture surface of the samples is calcite.

### 3.4. Microstructure of Self-Healing Mortar Sample

**Figure 10:**
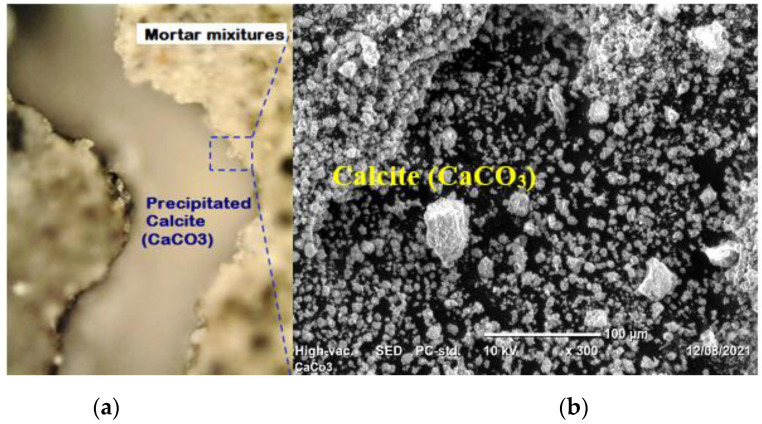
(**a**) Optical microscope image (×50 magnifications) for Self-Healing Mortar sample; (**b**) SEM image at the crack edge of the sample.

Optical microscope and SEM images (Figure 10) show the production of calcite along with the fracture location, implying that the mortar’s self-healing capacity is aided by bacterial activity via the generation of CaCO_3_ precipitates. These precipitated calcium carbonate crystal particles most likely filled some of the pores/voids inside of mortar samples, and also filled the fractures on the mortar surfaces. As a result, we anticipated an increase in the strength of SHM samples.

### 3.5. Thermal Analysis on Mortar Samples

**Figure 11:**
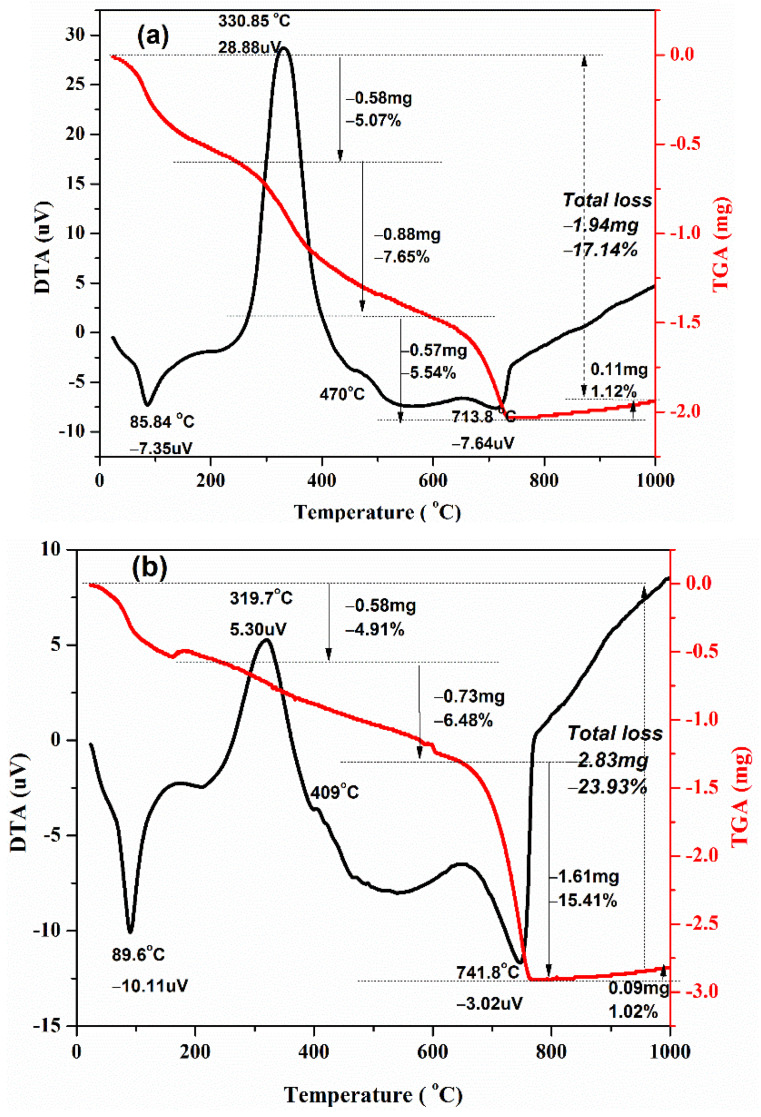
TGA and DTA curves for mortar samples: (**a**) conventional mortar; (**b**) self-healing mortar.

We used DTA-TGA on the hardened paste to forecast the thermal stability of conventional mortar and Self-Healing Mortar (SHM) samples respectively, as shown in Figure 11a,b. According to TGA data, both samples lost weight as the burning temperature increased, most likely owing to water loss, the breakdown of components, and the escape of volatile particles from mortars. The endothermic peak at 86 °C to 90 °C is caused by moisture loss from the samples. According to Peter C. Hewlett et al. [35], when the cement paste is heated, the water combined in the hydrated cement compounds other than calcium hydroxide is primarily lost below 350 °C, but the water combined in calcium hydroxide is virtually fully lost between 350 °C and 550 °C. The loss of water from cement at temperatures ranging from 350 to 550 °C may be utilized to calculate the calcium hydroxide concentration. Thus, our experimental results reveal that weight losses for conventional mortar and SHM samples from 25 °C to 330 °C are most likely due to water vaporization in the hydrated cement components. Exothermic peaks at 410 °C and 470 °C reflect dehydroxylation of the calcium-hydroxide phase, i.e., breakdown of Ca(OH)_2_ to CaO, generated by the hydration reaction of the cement phases, as previously discussed [36,37]. Figure 11a,b illustrate that the weight loss of the samples above 550 °C is most likely due to the decomposition of calcite CaCO_3_ to free CaO and CO_2_ (g), causing volatile matters to escape (decarbonization). Decarbonization reaction is highly endothermic. However, around 650 °C, small exothermic peaks have also appeared. This is probably due to the formation of phase which is caused by the reaction of free CaO and oxides in cement. According to our TGA investigation, the water loss of the SHM sample is less than that of conventional mortar. This is because the holes and spaces inside the SHM sample are most likely filled with precipitated calcium carbonate, which is generated by bacteria in the SHM and contributes to the sample’s compactness. This may be supported by the fact that the weight loss (due to CaCO_3_ decomposition on the temperature range 600 °C to 750 °C) of the SHM sample (15.41 percent) is greater than that of the conventional mortar sample (5.54 percent). It is further supported by the results of water absorption tests on conventional mortar and SHM samples.

### 3.6. Compressive Strength for Mortar Samples at Pre-Crack Development Time

**Figure 12:**
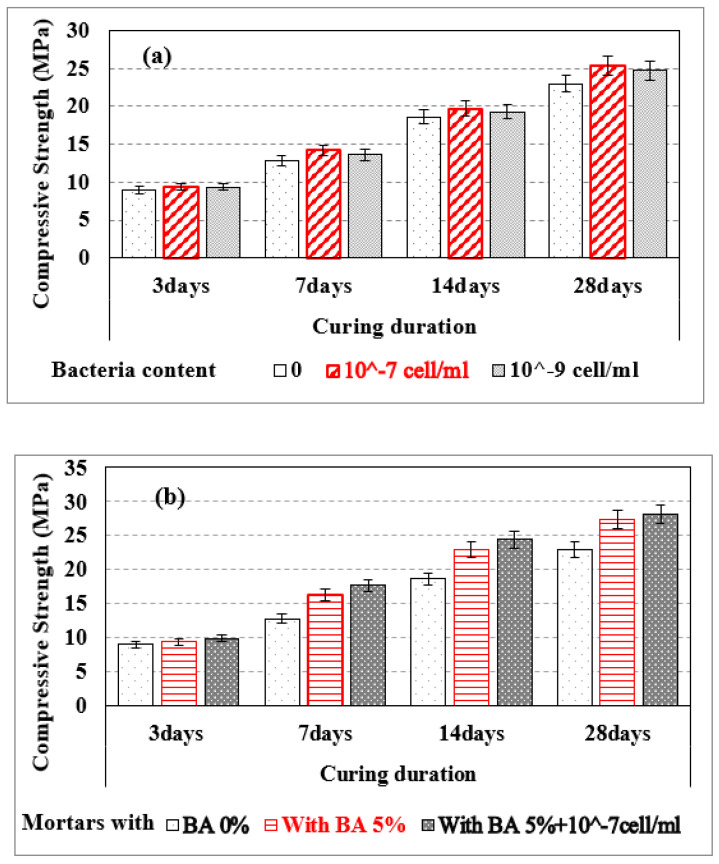
Compressive strengths of samples: (**a**) conventional mortar (BA 0%) and SHM with different bacteria cell/mol; (**b**) conventional mortar sample (BA 0%), SHM sample with 5% BA, and SHM with 5% BA w, Bacteria 10^−7^ cell/mL.

Figure 12 shows that the compressive strength of Self-Healing Mortar (SHM) samples with bacteria (10^−7^ or 10^−9^ cells/mL) was greater at 3, 7, 14, and 28 days compared to conventional mortar samples before crack formation (Figure 12a). For instance, SHM samples with bacteria 10^−7^ cells/mL and with 10^−9^ bacteria cells/mL have higher compressive strength, more 4.6% & 4.1% respectively, as compared to conventional mortar sample at 3 days of cure. Similarly, SHM samples with bacteria 10^−7^ cells/mL and with 10^−9^ bacteria cells/mL have higher compressive strength, more 9.1% & 6.9% respectively, as compared to conventional mortar sample at 28 days of cure.. This is due to precipitated calcite filling the micropores of the mortar samples and making them more compacted, as previously discussed [31,32]. A higher concentration of bacteria cells (10^−7^ cells/mL) causes more calcite to precipitate and fill the mortar’s micro-pores. As a result, the compressive strength of mortar samples was raised with the concentration of bacteria cells for 10^−7^ cells/mL. For 3, 7, 14, and 28 days of curing, SHM with bacteria at 10^−7^ cell/mL and 5% BA had better compressive strength than BA-blended mortars and conventional mortars (Figure 12b). For example, the compressive strength of SHM samples containing 10^−7^ cells/mL bacteria with 5% BA was 18.2% greater than that of conventional mortar samples after 28 days of hardening.

### 3.7. Flexural Strength of Mortar Samples

Samples with Table 2 compositions and sizes 160 mm × 50 mm × 50 mm are made and then cured for 7 and 28 days. Flexural strength mortar samples were assessed using ASTM C348-08 [38] before and after fracture development. All mortar specimens were subjected to a three-point bending test (3PBT) to determine their flexural strength. Before crack development, 5% C-BA blended Self-Healing Mortars (SHM + 5% BA) have higher flexural strength compared to conventional mortar and SHM at curing ages of 7 and 28 days. However, after the cracks healed, SHM has higher flexural strength (σF = 340 kPa) than SHM + 5% BA (σF = 100 kPa) at 28 days’ curing, as indicated in Table 3. The broken pieces of conventional mortars were not healed and did not withstand any applied force (σF = 0 kPa) at the post-crack period. The tendency to heal the cracks decreased significantly to reach almost none with the addition of a large quantity (≥10%) of bagasse ash in SHM samples, as did the usage of BA burnt at 300 °C, as did the usage of BA burnt at 300 °C. We anticipated that a large amount of BA replacement might not suitable for bacteria to survive and precipitate calcite. This might be a reasonable hypothesis to lower the fracture healing efficacy of mortars with the addition of a higher (≥10%) amount of BA. Thus, we prepared an SHM sample containing only 5% C-BA.

### 3.8. Water Absorption of Mortar Samples

**Figure 13:**
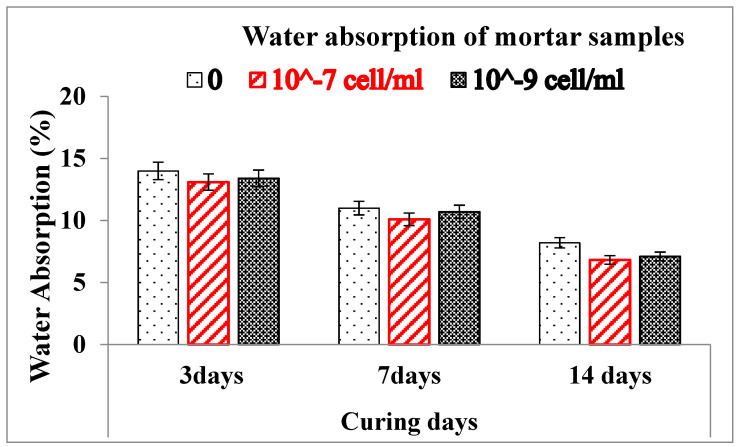
Water absorption of mortar samples: conventional mortar and Self-Healing Mortar.

Self-Healing Mortar samples have a lower water absorption value compared to conventional mortars as illustrated in Figure 13. This is most probably the precipitated calcium carbonate that might fill pore space inside the SHM samples, resulting in the compactness of the samples. SHM samples absorbed less water, resulting in a more impermeable mortar that is useful in boosting fracture strength, minimizing sulfate assault, and strengthening the air-void network. Figure 13 depicts the reduction in water absorption of conventional mortars with the addition of bacteria and with curing ages. Compared to the conventional mortar sample, SHM samples containing 10^−7^ cells/mL show a maximum water absorption reduction of 1.38% after 14 days, and the presence of bacteria produced a decrease in water absorption after 3, 7, and 14 days.

## 4. Conclusions

We successfully made conventional mortar samples, self-healing mortar samples, and self-healing mortar samples with bagasse ash. Surface fractures of up to 0.6 mm width were filled by the precipitated calcite in self-healing mortar samples. The healing efficiency of mortar was improved with the addition of bacteria 10^–7^/10^–9^ cells/mL and curing days. Cracks on mortar samples having bacterial concentrations of 10^–7^ cells/mL as well as 10^−9^ cells/mL were filled by calcite after 7 and 14 days of curing ages. However, the fracture healing effectiveness of mortars was reduced as control-burnt bagasse ash replacement increased. Self-healing mortar samples are thermally stable in the same way as conventional mortars. However, the weight loss of Self-Healing Mortar (SHM) is greater than that of conventional mortar at higher burning temperatures (up to 750 °C). The extra precipitated calcite in SHM samples started to decompose at a temperature greater than 600 °C, causing a greater weight loss (15.41%) in the SHM sample than the conventional mortal sample (5.54%). The compressive strength of SHM samples with bacteria at 10^−7^ cell/mL and with bacteria at 10^−9^ cells/mL was higher than the compressive strength of regular mortar at 3 days of curing ages. Similarly, at 28 days of curing, the compressive strength of SHM samples containing bacteria at 10^−7^ cells/mL and 10^−9^ cells/mL were 9.1% and 6.9% higher, respectively, compared to conventional mortar. At pre- and post-crack development times, the compressive strength of self-healing mortars was higher than that of conventional mortars. The flexural strength of 5% bagasse-ash-blended self-healing mortar sample was higher than conventional mortar before cracks developed in the samples. After samples disintegrated (at post-crack development time), SHM containing 5% bagasse ash healed the broken pieces itself, and exhibited advanced flexural strength (100 kPa) compared to conventional mortars (zero kPa) at 28 days of curing time. This is attributed to the precipitated calcium carbonate filling the pore spaces and fractures in self-healing mortars, whereas cracks in traditional mortars were not repaired by themselves.

## Figures and Tables

**Table 1:** Mixing proportions mortar samples with 1% calcium lactate [W/(Cement + BA) ≈ 0.53].

Samples	Curing Days	Cement (%)	C-Bagasse Ash (%)	Bacteria Conc. [Cell/mL]
1	3	95	5	10^−7^
2	7	95	5	10^−7^
3	14	95	5	10^−7^
4	28	95	5	10^−7^

**Table 2:** Mixing proportion of mortar samples (CM, SHM and SHM + CBA) for flexural strength. (cement + BA: sand = 1:3, calcium lactate 1.5%).

Samples	Cement (%)	Bacteria Conc. (Cell/mL)	Bagasse Ash (%)	Water (mL)
CM	100	0	0	170
SHM	100	1 × 10^−7^	0	170
SHM + BA	95	1 × 10^−7^	5	170

**Table 3:** Flexural strength (σF) of Conventional Mortars (CM), Self-Healing Mortars (SHM), and bagasse-ash-blended Self-Healing Mortars (SHM + C-BA) at 7 and 28 days of curing.

Mortar Samples	Pre-Crack Formation Period on Mortars		Post-Crack Formation Period on Mortars
	Flexural Strength (MPa) @7 Days	Flexural Strength (MPa) @28 Days	Flexural Strength (kPa) @28 Days
CM	2.82	3.71	0
SHM	3.48	4.53	340
SHM + 5% C-BA	3.51	4.97	100

## Data Availability

Whole of Data is available in manuscript itself.
